# Motion Analysis of Core Stabilization Exercise in Women: Kinematics and Electromyographic Analysis

**DOI:** 10.3390/sports11030066

**Published:** 2023-03-10

**Authors:** Kyeongjin Lee

**Affiliations:** Department of Physical Therapy, College of Health Science, Kyungdong University, Wonju 24764, Republic of Korea; kjlee@kduniv.ac.kr

**Keywords:** core stability, core exercises, pilates, electromyography, kinematics

## Abstract

As core stabilization exercise is essential for maintaining a stable spine and improving functional performance, understanding the activation of core muscles and the stabilization of the trunk and pelvis during such exercise is crucial. The purpose of this study was to investigate the muscle activation and stabilization of the lumbar–pelvic region during core stabilization exercise, with a specific focus on analyzing EMG and 3D motion kinematic data. The study aimed to understand how different tension settings on the reformer affect muscle activation and hip motion, as well as how these factors impact pelvic and trunk stability during the exercise. The reformer consists of a carriage that slides back and forth on rails, with springs providing resistance. The springs can be adjusted to vary the resistance level. Twenty-eight healthy women participating in this study were asked to perform ‘side splits’, a hip abduction exercise, on the reformer in both heavy and light tension settings. Activation of the internal oblique (IO), rectus abdominis (RA), multifidus (MU), costal lumbosacral (IL), gluteus medius (GM), and adductor muscles (AL) were measured using electromyography (EMG) and 3D motion. Kinematic data using an assay were also measured during exercise. GM, IO, and MU muscles were more active when heavy springs were used, and AL muscles were more active when light springs were used. Hip motion was more symmetrical when lighter springs were used with a greater range of hip motion. There was less pelvis and torso weight transfer and more torso and pelvis stability when the heavier springs were used. In this study, we confirmed that core stabilization exercise on an unstable surface activates the deep muscles of the abdomen and back and is effective for pelvic and trunk stabilization training.

## 1. Introduction

Low-back pain (LBP) is a highly prevalent disability that affects nearly 80% of people during their lifetime and is the leading cause of disability worldwide [1,2]. Approximately 40% of 9–18 years old in high- to low-income countries report having had LBP, while most adults present with LBP at some point [3,4]. LBP places large burdens on work disability, loss of wages and productivity, care seeking and medication use, and early retirement [5,6]. Most LBP cases are non-specific because its specific causes or nociceptive sources are rarely identified [7]. It can result from different abnormalities or diseases that are either known or unknown; thus, LBP is not a term for a disease but a symptom [7]. LBP is accompanied by activity limitations that increase with age [8]. It commonly accompanies pain in the lower limbs, and some portions of the population also present with associated neurological symptoms in their legs. Studies have suggested that LBP is a pain syndrome with a mixed form of nociceptive and neuropathic components [9]. People with LBP are also at a greater risk of developing physical problems in other body sites and/or mental illness than those without LBP [10].

LBP treatment primarily aims to relieve pain, restore normal function, and prevent chronicization [11,12]. Along with other treatment options, therapeutic exercise is one of the most cost-effective strategies for coping with LBP without invasive operations [11,12]. Exercises can help improve back extensor strength, mobility, endurance, and functional disability [13]. Exercises such as lumbar flexion and extension, walking, core, motor, lumbar stabilization, and bracing are common therapeutic exercises for LBP [14]. These exercises mainly focus on lumbar stabilization and core muscle strengthening [15]. One well-known lumbar stabilizing exercise uses a pressure biofeedback unit to indirectly monitor abdominal core activation during isometric abdominal contraction [16,17,18].

Core stabilization has gained popularity in rehabilitation and sports training, as its importance in preventing musculoskeletal injuries and enhancing performances has been established [19,20]. The stabilizing system of the spine depends on the complex interplay mechanisms of the spinal column, muscles, and neural control unit [21,22]. Reeves et al. [23] mentioned that stabilization incorporates maintaining balance in either static or dynamic conditions as the body moves for a specific task. Disturbances in one or more of these subsystems may lead to an abnormal range of motion and cause tissue injury, leading to LBP [23]. The ability to subsequently control the lumbar–pelvic complex at the neuromuscular level is vital for maintaining trunk posture, maximizing movement efficiency, and preventing injury [24]. Comerford and Mottram classified the muscles of the lumbar spine according to their region as local stabilizers, single-joint global stabilizers, and multi-joint global stabilizers [25,26]. Local stabilizers control segmental translation by increasing the segment stiffness [27,28,29]. Their activation is constant and independent of the direction of movement, functions in the feedforward system, and is activated earlier than the muscles that generate torque for the movement to maintain the static placement of the body segment [30,31]. Global stabilizers generate force to control the range of motion and depend on the direction of the movement [27,28,29,30,31]. All of these muscles work together with passive (osteoarticular ligamentous) structures of the spine and neural control systems to maintain lumbopelvic stabilization [21].

Several studies have suggested the importance of core stabilization in reducing pain and instability in LBP patients [14,27]. Atrophy of paraspinal muscles with increased fatigability and neuromuscular control deficits due to altered central processing may compromise lumbar spine stability in patients with LBP [32,33].

Recent studies have investigated the effects of core stabilization training by adopting proprioceptive exercises, task-based training, weight-shift training, and the use of pressure biofeedback units [14,34]. In particular, unstable surface training has been known to be more effective on core stabilization training compared to training on a stable surface [35]. This is because an unstable surface provides an unexpected environment and activates proprioception; thus, human balance and core stabilization can be effectively trained [35]. The training methods on unstable surfaces involve the use of various tools, such as a Swiss ball, BOSU, and balance pad, depending on the exercise purpose [35,36,37,38,39]. Cosio-Lima et al. [37] used a Swiss ball during unstable surface training and confirmed improvement in rectus abdominis and erector spinae muscle activation and overall balance. Desai and Marshall [38] also demonstrated increased muscle activation of the rectus abdominis, internal and external obliques, and erector spinae on electromyography (EMG) analysis after unstable surface-based core training. Imai et al. [39] also trained five types of core stabilization exercises in healthy adults and indicated better activation of the rectus abdominis, oblique, multifidus, and erector spinae muscles on unstable surface training compared to stable surface training. Although previous studies have demonstrated the effectiveness of unstable surface training compared to stable surface training, they have not been able to control for the degree of instability during training. The reformer is an equipment used in Pilates exercises that can control perturbation and resistance of the unstable surface called ‘carriage’ by adjusting the tension of the springs that are connected to the reformer body.

Therefore, the purpose of this study was to confirm muscle activation and stabilization of the lumbar–pelvic region by analyzing EMG and kinematic data for core stabilization exercise according to an unstable support surface using a reformer.

## 2. Materials and Methods

### 2.1. Subjects

Twenty-eight healthy women recruited online were included in the study. The exclusion criteria included subjects who presented with disturbances of balance due to orthopedic or neuropathic disorders, those with cardiopulmonary disorders, those who had any surgeries six months prior, or those with any other disabilities. This study was approved by the ethics committee of Kyungdong University. All experiments were conducted in a biomechanics laboratory located at a university in Seoul, Republic of Korea. The purpose and procedure of this study were explained to the participants prior to the experiment, and all subjects provided written informed consent. To determine the sample size of the study, G-power 3.19 computer software (Heinrich Heine University Düsseldor, Düsseldorf, Germany) was used to determine the study’s sample size. The significance level is 0.05, the statistical power is 0.95, and the effect size is 0.8. Twenty-three people were needed to compare heavy and light tension, and 28 participants were recruited considering dropout.

### 2.2. Core Stabilization Exercise

**Figure 1:**
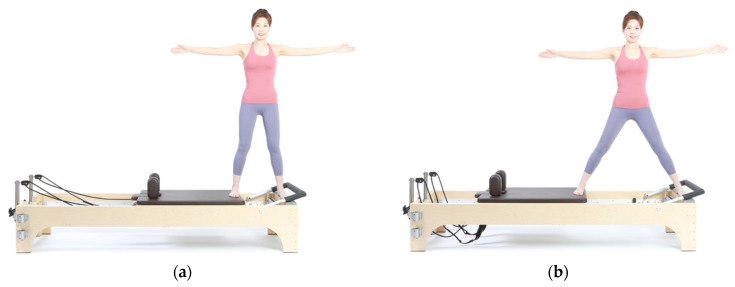
Side split while standing on the Pilates reformer: (**a**) starting position with neutral spine; (**b**) end position with carriage pushed away.

The subjects performed ‘side splits’, a hip abduction exercise using a Pilates reformer (V2 Max™ Reformer; Toronto, ON, Canada). The side split started by maintaining a neutral spine while standing on the reformer. One foot rested on the standing platform, while the other was placed on the edge of the carriage. Their legs were straight and parallel to each other. The scapulars were stabilized while both arms were straight, with shoulders abducted at 90° (Figure 1a). While maintaining the pelvis and trunk in position, the hip joints were abducted to push the carriage away. At this point, the weights of both legs were equally distributed, as the trunk maintained symmetry (Figure 1b). The subjects then return to their original position after maintaining the abducted position for 1 s. Two different resistances during the side splits were performed: one white spring (initial tension 1.0–1.5 kg, spring rate 0.05 N/mm +/− 5%) for light tension and a white and a blue spring (initial tension 1.4–1.8 kg, spring rate 0.11 N/mm +/− 5%) for heavy tension. Before the measurement, the choreography and precautions were explained to each participant. The subjects practiced three times before the trial. A total of 40 s was required to perform five repetitions, with each repetition comprising 8 and 3 s for the moving part, and 1 s for the holding part, with a series of motions back and forth. After five repetitions were performed for each of the two different spring tension settings, the sides of the legs were switched.

### 2.3. Kinetics and Electromyographic Analysis

To measure muscle activation, surface EMG (Ultium EMG^®^, Noraxon, AR, USA) was used with dual EMG wet gel electrodes (single electrode T246H, SEEDTECH, Cheonan-si, Republic of Korea). We ensured that all electrodes were below 70 K by shaving and abrading the skin with alcohol. The electrodes were placed on the following areas of the muscles assessed: the internal oblique (IO), 2 cm medially and inferiorly; the anterior superior iliac spine (ASIS); rectus abdominis (RA), 3 cm from the midpoint line of the umbilicus; iliocostalis lumborum (IL), 2 cm from the spinous process of the first lumbar (L1) vertebra; multifidus (MU), 3 cm from the midpoint line from the spinous processes of the L1 to L5 vertebrae; gluteus medius (GM), two electrodes parallel over the proximal third of the distance between the iliac crest and the greater trochanter; and the adductor longus (AL), two electrodes on the medial aspect of the thigh, 4 cm oblique line away from the pubis. In the study, we applied a bandpass filter to the raw surface EMG signals to eliminate any unwanted frequencies that could interfere with their analysis. The bandpass filter was set between 20 and 400 Hz, allowing the signals to pass through a range of frequencies typically associated with muscle activity. A bandpass filter is a type of filter that allows signals within a specific frequency range to pass through while blocking signals outside that range. In sEMG analysis, a bandpass filter is used to eliminate any unwanted frequencies that could interfere with the analysis of muscle activity. The sample rate was 2000 Hz, and the raw data were processed as root mean square using a window of 60 ms after rectification and smoothing. Normalization was performed using MVIC to account for individual and experimental differences in signal amplitude. Normalization aims to create a consistent baseline for comparing EMG signals across different trials, muscles, or individuals. Each subject exerted maximal effort during each MVIC. MVIC contracted three times for 5 s as 1 set, and the value was used as the average of 3 sets. A 10 min break was given between each set. During the 5 s contraction, the average values for 3 s, excluding 1 s back and forth, were used as the MVIC value. EMG values measured during movement were expressed as a percentage of this MVIC value [40].

Kinematic data were measured by placing ProReflex motion capture cameras (Qualisys AB, Gothenburg, Sweden) around the participants while they performed side-split exercises on the reformer. Each camera projects infrared light, and the markers are retroreflected back to the cameras. The camera captured the markers at 200 Hz and then tracked the movement of the markers in 3D to process with the Qualisys motion capture system (Qualisys, Qualisys AB, Gothenburg, Sweden). Reflective markers (6.5 mm diameter) were placed in the following anatomical landmarks to define the head, arm, trunk, pelvis, and leg rigid bodies: the top of the head, above the left and right external auditory meatus; left and right acromions; on the lateral epicondyles of the elbows; on the wrists; on the spinous processes of C7, T3, T7, T12, L5, sacrum, ASIS, iliac crests, posterior superior iliac spines (PSIS) and greater trochanters; lateral and medial condyles of the knees; and on the lateral malleolus of the ankles. The movement of the carriage was captured by placing four markers at both ends of the footbar and the carriage. The tracked coordinates of the markers were transcoded into 3D motion images using Qualisys Track Manager (Qualisys, Qualisys AB, Gothenburg, Sweden), and the movement of the rigid bodies was analyzed using MATLAB software (Matlab 2021a, Mathworks Inc., Natick, MA, USA). The signal was processed using a low-pass Butterworth filter in the motion capture software [41].

Trunk stability was assessed by calculating the cumulative distance of the trunk’s center of mass. The trunk segment was defined using acromion and iliac spine markers on both sides, and the center of the segment was used to calculate the distance. Pelvic stability was assessed by calculating the cumulative distance from the pelvic center of mass. The pelvic segment was defined using the ASIS and PSIS markers on both sides, and the center of the segment was used to calculate the distance, as described above. The symmetry between the hip joints was calculated as the ratio of the abduction angles of each leg (Formula (1)).

(1) $$\mathrm{hip}\mathrm{joint}\mathrm{symmetry}=\frac{\mathrm{Anchored}\mathrm{leg}\mathrm{angle}}{\mathrm{Sliding}\mathrm{leg}\mathrm{angle}}$$

### 2.4. Statistical Analysis

All data were normally distributed when examined using the Shapiro–Wilcoxon test. Data are expressed as mean and standard deviation. Muscle activation and kinematic data among the different tension levels were compared using a paired t test. Pearson’s correlation analysis was performed to analyze the correlation between kinematic data and muscle activation data [42,43]. IBM SPSS (version 23.0; IBM Corporation, Armonk, NY, USA) was used for all the statistical analyses. Statistical significance was set at *p* < 0.05.

## 3. Results

The general characteristics of the study participants are presented in Table 1. A total of 28 healthy women were recruited for the study. The age of the subjects ranged from 20 to 29 years old.

### 3.1. Muscle Activity during Side Split Exercise

Muscle activation depending on the tension settings is described in Table 2. The GM was activated 3.46 times greater when the tension was set to heavy than when it was set to light (*p* < 0.05). The AL was activated 5.98 times greater when the tension was set to light than when it was set to heavy (*p* < 0.05). IO and RA muscle activation showed statistical significance when the tension was light and heavy (*p* < 0.05). MF and IL muscle activation showed statistical significance when the tension was light and heavy (*p* < 0.05).

### 3.2. Kinematic Analysis of the Side Split Exercise

The kinematic data from the motion analysis during the side-split exercise is presented in Table 3. The hip abduction angles of both legs were greater when trained using light springs than when trained using heavy springs (*p* < 0.05). The hip moved more symmetrically when the light springs were used over the heavy springs (*p* < 0.05). The trunk and pelvis were more stable when heavy springs were used versus light springs (*p* < 0.05).

### 3.3. Correlation between Muscle Activation and Kinematic Data

The correlations between muscle activation and kinematic data are presented in Table 4. During heavy tension, hip joint symmetry, IO, RA, MU activation, and MU/IL ratio showed moderate correlation. During trunk stability and IO activation, IO/RA ratio activation showed strong correlation, whereas RA, MU activation, and MU/IL ratio showed moderate correlation. During pelvic stability and IO activation, IO/RA ratio showed strong correlation, whereas the other variables showed moderate correlation. In light tension, trunk stability, RA activation and IO/RA ratio showed moderate correlations, whereas IO activation showed strong correlation. During pelvic stability, RA activation and IO/RA ratio showed moderate correlations, whereas IO activation showed strong correlation.

## 4. Discussion

Muscles that contribute to the spinal stabilization system include local muscles, which provide segmental stability, and global muscles, which provide mobility [44]. The primary segmental muscles are the MU from the lower back and the IO and transverse abdominis muscles from the abdomen. Isolated training of these stabilizers has been emphasized and studied for the prevention and rehabilitation of low back injuries. One training method for stabilizing muscles is to exercise on unstable surfaces [39,45]. This form of exercise is thought to boost core muscle use, resulting in the activation of stabilizers [46]. This study analyzed muscle activity of the abdomen, lower back, and kinematic data using a 3D motion analysis system during core stabilization exercise on an unstable support surface. The main result of this study was that the muscle activity according to the spring tension was compared. Compared to light tension, the muscle activities of GM, IO, and MU were higher under heavy tension, confirming that the core muscles were more activated. It was confirmed that AL was more activated. All kinematic data (hip abduction of the anchored leg and hip abduction of the sliding leg movement, hip joint symmetry, trunk stability, and pelvic stability) showed significant differences between spring tensions.

Many studies have highlighted the activation of core muscles using Pilates [47,48]. Barbosa et al. [49] compared subjects with and without Pilates experience to examine differences in abdominal muscle activation. Subjects with Pilates experience maintained a high level of transverse abdominis/IO activation. However, those without experience reached only half the level of the experienced group; moreover, none in this group reached the targeted goal level. The differences between these two groups show how Pilates can effectively activate deep abdominal muscles. Lee’s [41] study also confirmed that experienced Pilates practitioners effectively activated the abdominal and lower back core muscles and improved pelvis and trunk stability compared to non-experienced subjects. In addition, the better the trunk stability is maintained, the greater the mobility and the more accurate the movement. Gala-Alarcón et al. [50] studied the effect of Pilates on the abdominal wall and lumbar MU muscles using ultrasound evaluation. They assessed the thickness of the transverse abdominis, IO, external oblique, RA, and MU muscles after one year of Pilates and found an increase in the thickness of the transverse abdominis, IO, and MU muscles. Alves et al. [51] adapted Pilates as a therapeutic method for non-specific low back pain patients and healthy participants for 16 sessions spaced in eight weeks. They observed activation of the lumbar extensor, IO, and transverse abdominis muscles using EMG. The visual analog scale, positive rate from the prone instability test, abnormal movement rate, trunk flexibility, and fear avoidance-related questionnaire were also measured. Non-specific LBP patients showed improved extension strength and activation time to the same degree as healthy subjects, with a decreased visual analog scale, positive rate, and abnormal movement rate. Activation patterns were restored to levels in the control group. These results show that Pilates is effective in preventing and alleviating musculoskeletal diseases by affecting deep muscle activation. Panhan et al. [48] reported different activations of trunk muscles during exercises on different-sized supporting bases. Activation of the RA and IO muscles represent the activation of mobilizers and stabilizers, respectively. They revealed that a decrease in the supporting base increased muscle activation, similar to the results of our study, which provided an unstable surface.

In our study, the moving feature of the carriage was regarded as an unstable surface, and core muscle activation was explored. The side splits activated the core muscles of the lower back and abdomen, with the heavy tension setting increasing their activation compared to the light tension setting. Superficial muscle activation did not present statistically significant differences and only presented an overall low activation. Our results suggest that side splits effectively activate core muscles, whereas the heavy tension setting further amplifies core muscle strengthening.

Moreover, observing the abductors and adductors of the hip joints during side splits is interesting. Light tension conditions activated the adductors 3.46 times more than heavy tension conditions, while heavy tension conditions activated the abductors by 5.98 times more. Therefore, we confirmed that a light tension setting is efficient in activating adductors, whereas a heavy tension setting is more efficient in activating abductors.

The symmetry of the body during side-splitting is another essential aspect of movement. In particular, trunk stability and pelvic stability in heavy tension showed strong negative correlations with IO, RA, MU, IORA ratio, and MUIL ratio. A stably controlled local muscle can create and maintain an ideal alignment of the trunk and pelvis; therefore, it is thought that unnecessary excessive activity of the global muscle is reduced. In contrast, under light tension, trunk and pelvic stability showed a negative correlation with IO, RA, and IO/RA ratio, but there was no correlation with MU, IL, and MU/IL. This is because the abdominal muscles acted as a stabilizer, but the back muscles did not act as a stabilizer; thus, trunk stability and pelvic stability could not be maintained.

Hip joint symmetry was better when light tension was applied, but the range of motion of the hip joint was large. To control the symmetry of the hip joint, the adductor muscle played a controlling role in light tension, and the abductor muscle played a controlling role in heavy tension. In the case of heavy tension, the movement of the anchored leg was greater than that of the sliding leg, but the activity of both abductors did not show a difference; therefore, it seems that it did not play a role in controlling the symmetrical movement. Side split was shown to enhance the stability of the trunk and pelvis by eliciting higher activation of local muscles when heavy tension was used. To prevent lumbar and pelvic diseases by stabilizing the trunk, deep muscle exercises have been recommended. However, training for isolated contraction of deep muscles requires expensive equipment. It is difficult to recognize local muscle contraction without feedback equipment, and training using feedback equipment has limitations in that the exercise method is simple. Side split is considered an effective intervention method for core exercises that can induce local muscle activity by controlling tension resistance without the special recognition of local muscle contraction.

This study has several limitations that should be considered when interpreting the results. First, the study only included healthy women; thus, the results may not be generalizable to other populations, such as men, older adults, or people with certain health conditions. Second, the study did not control for the participants’ fitness levels or prior experience with the exercise, which may have influenced their muscle activation patterns and performance. Third, the study only used EMG and 3D motion kinematic data to measure muscle activation and movement patterns. Other measures, such as force plate measurements, may have provided additional information on muscle activity and functional performance. Finally, the study was cross-sectional, which means that the researchers were only able to observe the participants at one point in time. This makes it difficult to establish cause-and-effect relationships between the variables studied. Therefore, further research involving randomized clinical trials is needed to fully understand the benefits and optimal strategies for core stabilization exercise.

## 5. Conclusions

In this study, the relationship between muscle activity and quality of movement of the lumbar and abdominal muscles during a side-split exercise on an unstable surface was investigated. It was confirmed that the heavy spring tension activated the abdominal and lower back deep muscles more effectively than the light tension, thereby maintaining the stability of the pelvis and trunk. For rehabilitation, it is effective to apply light tension step by step, while the heavy tension is effective for strengthening the core muscles. Pilates-based core stabilization exercise induces activation of the local muscle and is considered an effective method for trunk stabilization.

## Figures and Tables

**Table 1:** General characteristics of the participants.

	Mean (±SD)
Age (years)	22.13 (±1.36)
Height (cm)	162.69 (±5.45)
Mass (kg)	53.25 (±7.22)
Body mass index (kg/m^2^	20.03 (±1.57)

SD, standard deviation.

**Table 2:** Muscle activation depending on the different tension settings (μV).

		Light	Heavy	t	*p*
Gluteus medius	Right (%)	7.97 ± 4.37	27.7 ± 18.58	3.58	0.002
	Left (%)	8.71 ± 4.11	30.12 ± 13.68	5.192	0
	Total (%)	8.34 ± 2.95	28.91 ± 13.46	5.172	0
Adductor longus	Right (%)	27.88 ± 10.82	4.44 ± 3.97	7.049	0
	Left (%)	29.93 ± 12.33	5.23 ± 3.03	6.74	0
	Total (%)	28.9 ± 10.46	4.83 ± 3.03	7.659	0
Abdominal muscle	IO (%)	28.44 ± 17.71	33.42 ± 13.95	0.765	0.452
	RA (%)	10.07 ± 12.45	9.67 ± 10.06	0.087	0.932
	t	5.491	6.006		
	*p*	0	0		
	IO/RA (ratio)	10.9 ± 14.19	13.74 ± 16.24	0.456	0.653
Low back muscle	MF (%)	21.31 ± 16.67	19.35 ± 6.94	0.376	0.71
	IL (%)	8.58 ± 5.36	7.56 ± 3.84	0.534	0.599
	t	2.404	5.146		
	*p*	0.035	0		
	MF/IL (ratio)	3.35 ± 3.07	3.35 ± 2.27	0.007	0.994

Values are expressed as mean ± standard deviation. IO, internal oblique; RA, rectus abdominis; MU, multifidus; IL, iliocostalis lumborum.

**Table 3:** Kinematic analysis depending on the different tension settings.

Variables	Light	Heavy	t	*p*
Hip Abduction of Anchored leg (degree)	15.42 ± 5.22	12.39 ± 3.32	5.672	0.000
Hip Abduction of Sliding Leg (degree)	13.25 ± 4.41	9.76 ± 2.90	6.335	0.000
Hip joint Symmetry	1.20 ± 0.18	1.45 ± 0.43	4.811	0.000
Trunk Stability (mm)	2518.46 ± 890.05	1869.44 ± 647.20	8.331	0.000
Pelvic Stability (mm)	2515.28 ± 898.80	1828.94 ± 609.87	8.398	0.000

Values are expressed as mean ± standard deviation.

**Table 4:** Correlation between muscle activation and kinematic data.

		Hip Joint Symmetry	Trunk Stability	Pelvic Stability
Heavy	IO	−0.527 ∗	−0.776 ∗	−0.802 ∗
	RA	−0.402 ∗	−0.416 ∗	−0.443 ∗
	IO/RA	−0.201	−0.722 ∗	−0.754 ∗
	MU	−0.459 ∗	−0.558 ∗	−0.507 ∗
	IL	0.121	0.232	0.301 ∗
	MU/IL	−0.462 ∗	−0.653 ∗	−0.627 ∗
Light	IO	−0.343	−0.727 ∗	−0.792 ∗
	RA	−0.197	−0.608 ∗	−0.543 ∗
	IO/RA	−0.124	−0.663 ∗	−0.679 ∗
	MU	−0.294	−0.204	−0.305
	IL	−0.238	−0.168	−0.232
	MU/IL	−0.258	−0.297	−0.324

IO, internal oblique; RA, rectus abdominis; MU, multifidus; IL, iliocostalis lumborum. * *p* < 0.05.

## Data Availability

Not applicable.
